# A Keyword-Enhanced Approach to Handle Class Imbalance in Clinical Text Classification

**DOI:** 10.1109/JBHI.2022.3141976

**Published:** 2022-06-03

**Authors:** Andrew E. Blanchard, Shang Gao, Hong-Jun Yoon, J. Blair Christian, Eric B. Durbin, Xiao-Cheng Wu, Antoinette Stroup, Jennifer Doherty, Stephen M. Schwartz, Charles Wiggins, Linda Coyle, Lynne Penberthy, Georgia D. Tourassi

**Affiliations:** Computational Sciences and Engineering Division, Oak Ridge National Laboratory, Oak Ridge, TN 37830 USA; Computational Sciences and Engineering Division, Oak Ridge National Laboratory, Oak Ridge, TN 37830 USA; Computational Sciences and Engineering Division, Oak Ridge National Laboratory, Oak Ridge, TN 37830 USA; Computational Sciences and Engineering Division, Oak Ridge National Laboratory, Oak Ridge, TN 37830 USA; College of Medicine, University of Kentucky, Lexington, KY 40536 USA; School of Public Health, Louisiana State University Health Sciences Center, New Orleans, LA 70112 USA; Rutgers Cancer Institute of New Jersey, New Brunswick, NJ 08901 USA; Huntsman Cancer Institute, University of Utah, Salt Lake City, UT 84132 USA; Epidemiology Program, Fred Hutchinson Cancer Research Center, Seattle, WA 98109 USA; University of New Mexico, Albuquerque, NM 87131 USA; Information Management Services Inc., Calverton, MD 20705 USA; National Cancer Institute, Bethesda, MD 20814 USA; National Center for Computational Sciences, Oak Ridge National Laboratory, Oak Ridge, TN 37830 USA

**Keywords:** Machine learning, natural language processing, medical information systems

## Abstract

Recent applications of deep learning have shown promising results for classifying unstructured text in the healthcare domain. However, the reliability of models in production settings has been hindered by imbalanced data sets in which a small subset of the classes dominate. In the absence of adequate training data, rare classes necessitate additional model constraints for robust performance. Here, we present a strategy for incorporating short sequences of text (i.e. keywords) into training to boost model accuracy on rare classes. In our approach, we assemble a set of keywords, including short phrases, associated with each class. The keywords are then used as additional data during each batch of model training, resulting in a training loss that has contributions from both raw data and keywords. We evaluate our approach on classification of cancer pathology reports, which shows a substantial increase in model performance for rare classes. Furthermore, we analyze the impact of keywords on model output probabilities for bigrams, providing a straightforward method to identify model difficulties for limited training data.

## I Introduction

THE National Cancer Institute’s (NCI) Surveillance, Epidemiology, and End Results (SEER) program works with cancer registries to extract key cancer characteristics from healthcare records to create national estimates of cancer incidence. A key step in this process is the extraction of tumor characteristics including site, subsite, and histology, from electronic pathology reports. The reports provide a rich source of information to track diagnoses, treatments, and outcomes. However, data in current registries is primarily in the form of unstructured text, making automatic information extraction difficult [1]. To overcome the challenges associated with unstructured text, previous work has employed deep learning models for document classification with promising results [2]–[4]. Although deep learning approaches have been successful, the class imbalance inherent in registries’ datasets continues to be a key challenge to training robust production models.

For a given training set, class imbalance occurs when a small subset of classes occupies a large fraction of the samples. For example, in pathology reports, common cancer sites such as breast or lung will occupy a greater portion of the training data than relatively rare cancer sites such as larynx [5]. The distribution of classes in a training set is useful information for a classification model, providing an important signal to the model on how likely a class is to occur independent of any information from the text. However, for rare classes, the compounding issues of long sequences of raw text along with few training samples can lead to over-fitting and subsequently poor model performance during testing or production [6], [7].

The problem of class imbalance in training data has been addressed previously through several different approaches [6]–[14]. For text processing and classification, one commonly used strategy involves altering the training set through oversampling, undersampling, or synthetic data generation to boost model performance [7], [13], [14]. A similar approach is to introduce class specific weights to the loss function to prioritize rare classes during training [6]. Although introducing class weights can indeed be a useful approach, it cannot in principle overcome the issues associated with over-fitting due to few training samples. For example, in the case of a single training sample for a given class, the input data may consist of a sequence of thousands of tokens. Furthermore, many combinations of those tokens may only occur for that class. Class weights may make the prediction of the rare class more likely by the model, but the change in weights does not improve the ability of the model to selectively identify meaningful segments out of the larger input sequence that robustly describe the class. Sampling methods are similarly hindered by a lack of diversity in the training data for rare classes.

Here, we present a different approach to addressing class imbalance by incorporating keywords into model training. In order to motivate the need for keywords, we utilize a widely-used model for clinical text classification [3], [4], [15], [16]. After model training, we find the highest scoring bigrams associated with each class. For well-represented classes, the trained model is able to identify short segments of text that represent the class, however, for rare classes few keywords or phrases are found by the model, suggesting over-fitting. To overcome the difficulties associated with limited training data for rare classes, we assemble a set of representative keywords for each class. The keywords are then used as training data alongside the raw text during each training batch.

To test our approach to boost performance on rare classes in clinical text, we consider the classification of pathology reports for cancer site, subsite, and histology. Keywords for each class within a respective task are assembled using two different methods with an increasing level of automation: (1) concept unique identifiers (CUI) extraction, (2) normalized pointwise mutual information (NPMI) ranking. Both approaches show a boost in the macro F1 score for tasks with class imbalance. Unlike class weights, the boost in performance for rare classes for our approach does not compromise performance for well-represented classes. Our results show that adding keywords to model training provides a straightforward way to improve production applications of deep learning for healthcare text data.

## II Methods

### A. Adding Keywords to Model Training

In this work, we are motivated by the performance difficulties of deep learning models on rare classes for clinical text classification [3] to propose a strategy for incorporating keywords into model training. Our strategy has two main components: generating a set of keywords associated with each class, and updating model training to include keywords.

For keyword generation, we consider two approaches: (1) extract keywords from external data sources, (2) extract keywords using statistics from the training corpus. For approach (1), we adopted external knowledge sources from the cancer epidemiology domain. The NCI thesaurus (NCIt) [17] provides reference terminology for medical concepts and vocabulary by the National Cancer Institute (NCI). The NCIt provides preferred terms, synonyms, research codes, and information for clinical research and administrative activities. We employed the table from the NCIt that lists the classification codes of cancer site and histology defined by the International Classification of Diseases for Oncology, 3rd edition (ICD-O-3) and their corresponding NCI thesaurus and NCI metathesaurus codes (concept unique identifiers, CUIs). We then identified keywords associated with the ICD-O-3 from the “concept names” listed in the Unified Medical Language System (UMLS) [18] CUI dictionary.

Although authoritative external knowledge sources are very useful, such sources are likely not available for many text classification applications. Therefore, we also considered extracting keywords using statistics from the training corpus. Specifically, we used normalized pointwise mutual information (NPMI) [19], [20] to rank unigrams and bigrams for each class. Here, we considered each token and class as a binary random variable (i.e. present or not present) for each document in the training corpus. The normalized pointwise mututal information between a token *x* and a class *y* is then given by:

(1) $$\text{NPMI}=\frac{-1}{\log\left(p\left(x,y\right)\right)}\log\left(\frac{p\left(x,y\right)}{p\left(x\right)p\left(y\right)}\right)$$

where each probability is estimated using a simple count of the occurrences of a given token and/or class divided by the total number of training documents. We then retained the top 10 unigrams and bigrams by NPMI for each class to use as keywords in model training.

The assembled keywords, either using CUIs or NPMI, consist of multiple short segments of text associated with each class. To incorporate the keywords into training, we sample from the assembled segments during each mini-batch. The loss is then calculated for the samples and added to the standard cross entropy loss for the documents. Keyword sampling introduces three hyperparameters: the number of classes sampled (*N*_*C*_ held fixed at 128, equal to batch size for pathology reports), the number of keyword segments sampled per class (*K*), and the weighting of keyword loss (*α*). Therefore, a given training update has the following steps:

1. Calculate cross entropy loss from the given mini-batch of the training samples (*L*_*docs*_).
2. Randomly sample max(*N*_*C*_,*C*) classes from the total number number of *C* classes for the given task
3. From each of the selected *N*_*C*_ classes, randomly sample *K* keyword segments (e.g. CUIs, bigrams, and/or unigrams)
4. For each unique class, join the *K* sampled keyword segments into a single document, resulting in a batch of *N*_*C*_ keyword documents
5. Calculate cross entropy loss from the keyword documents (*L*_*key*_)
6. Perform back-propagation based on the weighted sum of the loss from the training samples and the keyword documents (*L* = *L*_*docs*_ + *αL*_*key*_)

To determine the hyperparameters, a simple scan was done for the number of keyword samples per class (*K*) and the weighting of keyword loss (*α*). We first fixed *α* at 1.0 and varied *K* ∈ 1, 5, 10, 20.A value of *K* = 5 gave the best results in terms of a sum of micro/macro F1 across tasks with CUI and NPMI keywords. We then varied *α* ∈ 0.25, 0.5, 1.0, 2.0 with *α* =1 giving the best results. Therefore, *K* =5 and *α* =1 are used for all reported results unless otherwise specified. The full micro and macro F1 results for all parameters tested can be found in Tables VII and VIII.

### B. Class Weights

For comparison with our proposed keyword strategy, we used class weights in the loss function to improve model performance on rare classes. The class weights (*w*_*i*_) were determined based on the logarithm of the inverse class frequency using the following equation:

(2) $$w_{i}=\log\left(\frac{\mu \sum_{i}c_{i}}{c_{i}}\right)$$

where *c*_*i*_ is the number of times class *i* occurs in the training corpus and *μ* is a hyperparameter. Class weights were not allowed to be less than 1. For the value of *μ*, we used the following: 0.05, 0.15, 1.0. All values tested gave similar results (in relation to the keyword strategy); *μ* = 0.15 was used for all reported results unless otherwise specified. The full micro and macro F1 results for all parameters can be found in Table IX.

### C. Datasets

The data consists of cancer pathology reports obtained from the Louisiana Tumor Registry (LTR), Kentucky Cancer Registry (KCR), Utah Cancer Registry (UCR), New Jersey State Cancer Registry (NJSCR), and Seattle Cancer Registry (SCR) of the SEER Program.^1^

We determined truth labels of the cancer pathology reports based on the Cancer/Tumor/Case (CTC) database, which stores all diagnostic, staging, and treatment data for reportable neoplasms in the SEER Data Management System (SEER*DMS). We consider the International Classification of Diseases for Oncology [21], Third Edition (ICD-O-3) coding convention for labeling the cases. The following 3 tasks were used for model training: cancer site, subsite, and histology. The study was executed in accordance to the institutional review board protocol DOE000619, approved by Central DOR Institutional Review Board on April 6, 2021 (initial approval on September 23, 2016).

To determine the impact of rare classes on model performance, we assembled two datasets from the cancer pathology reports. The development dataset, which was used in all reported results unless otherwise specified, consisted of 177,185 pathology reports from KCR and LTR. The production dataset, contained 4,404,942 pathology reports gathered from all 5 registries, and was used to test the benefits of our approach in a large-scale production setting. Statistics for both datasets can be found in Tables I–II.

### D. Model Architecture and Parameters

Although our proposed strategy does not depend on a particular model, a specific architecture is needed to generate results. Here, we selected a word-level Convolutional Neural Network (CNN) tailored to extract information from a cancer pathology data corpus [3], [4], [15]. Although the model architecture is relatively simple, it is still widely used for biomedical text applications and produces near state-of-the-art results [3], [16].

Our CNN uses trainable word embeddings of size 300 that are initialized using Word2Vec pretraining on our train set. These are fed into three parallel 1D convolution layers with 300 filters each and window sizes of 3, 4, and 5 consecutive words. The convolution layer outputs are fed into a maxpool-over-time layer and concatenated, resulting in a document embedding vector of size 900. This final document embedding is fed into a softmax layer for classification. We train a separate model for each of our three classification tasks - site, subsite, and histology. We train with batch size 128 using the Adam [22] optimizer with learning rate 1E-4; training stops when loss does not improve on the validation set for 5 consecutive epochs. All models are trained using PyTorch [23] and a Tesla P100 GPU.

### E. Performance Metrics

We applied micro- and macro-averaged F1 scores for performance metrics:

(3) $$\text{Precision}=\frac{TruePositive}{TruePositives\;+\;FalsePositives}$$

(4) $$\text{Recall}=\frac{TruePositives}{TruePositives\;+\;FalseNegatives}$$

(5) $$\text{Micro F}1=2*\frac{Precision\;\times \;Recall}{Precision\;+\;Recall}$$

(6) $$\text{Macro F}1=\frac{1}{\left|C\right|}\sum_{C_{i}}^{C}F1\left(C_{i}\right)$$

In (6), *F*1(*C*_*i*_) is the F1 score within class *i*, and |*C*| represents the total number of classes in the dataset. Calculations were performed using the f1_score function from Scikit-learn [24].

F1 scores are widely accepted means of scoring for information extraction from cancer pathology reports [2]–[4]. The macro-averaged F1 is particularly useful for assessing severely imbalanced data corpus because it equally weighs the performance on each class including the rare classes. In addition to F1 scores, we determined the test accuracy for samples according to the number of training samples present. This enabled us to better isolate the impact of keywords on model performance.

We also assessed the performance of trained models by evaluating a given model on all possible bigrams from the training text. Bigrams were assembled using a sliding window of size 2 (i.e. no skip grams were added). Each bigram was then padded to the minimum document length necessary for the model (i.e. 5) and scored. The top bigrams were determined for each class based on the model score.

## III Results

### A. Impact of Keywords on F1 Scores

**Fig. 1.**
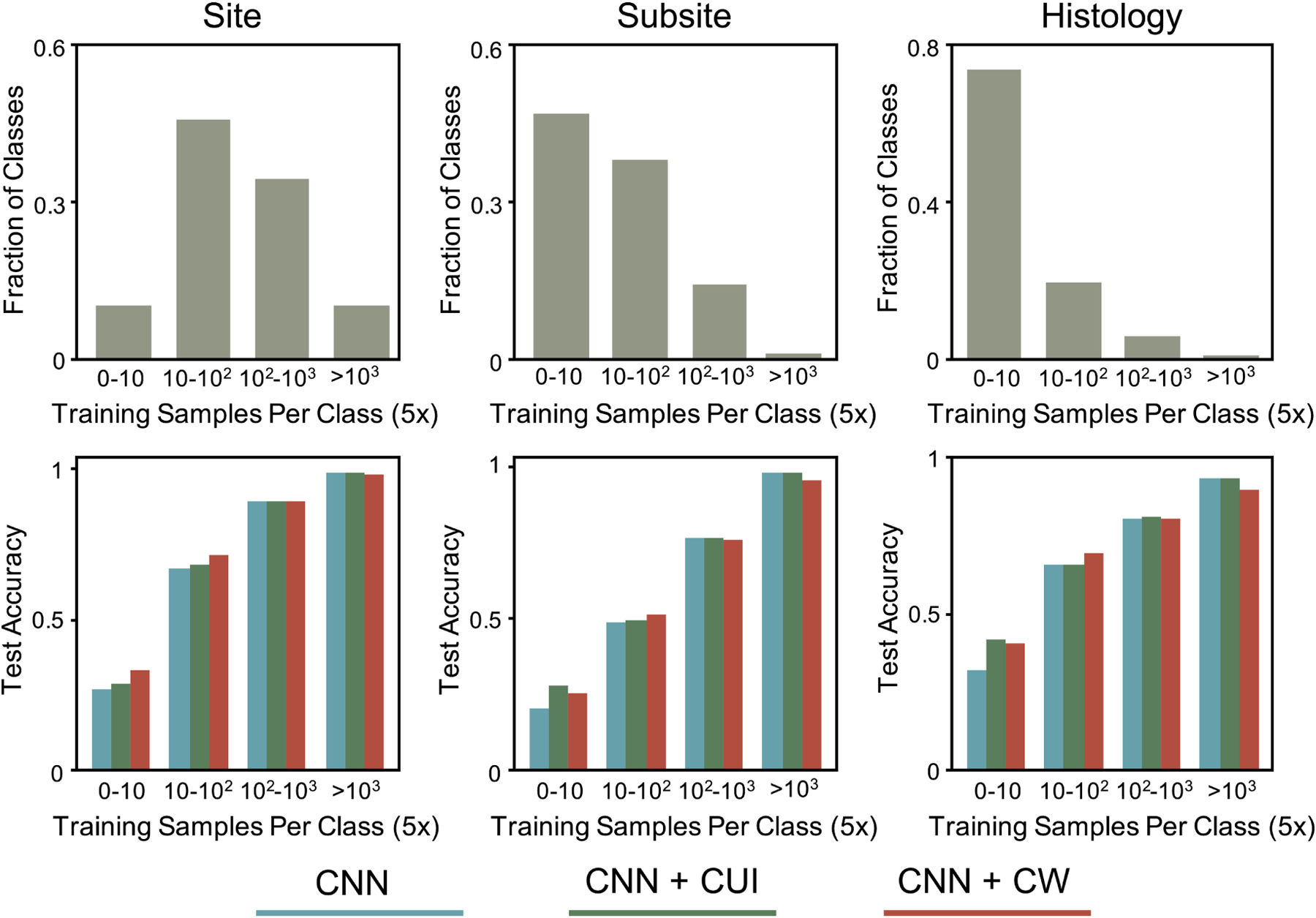
CNN model performance on the development dataset for three different tasks (site, subiste, histology). First row shows fraction of classes vs training samples per class. Second row shows test accuracy vs training samples for the baseline CNN model (blue), CNN + CUIs (green), and CNN + Class Weights (red). The x-axis for all plots is scaled by a factor of 5 (i.e. the intervals are 0–50, 50–500, 500–5000, and >5000 training samples).

As shown in Table III, the added keywords in the form of CUIs, improves on both micro and macro F1 scores for all three tasks (site, subiste, and histology) compared to the standard CNN model. The largest gain in macro F1 is realized for the task (histology) with the largest fraction of rare classes, as shown in Fig. 1. The results for the subsite and site tasks show that the benefit of keywords decreases as the fraction of rare classes decreases.

An important comparison to the keyword results is the performance with class weights (CW) added to the model. As shown in Table III, class weights are indeed capable of boosting performance on under-represented classes, resulting in an increase in macro F1. However, the increase in macro F1, is accompanied by a decrease in micro F1. Depending on the application, a drop in micro F1 may not be acceptable.

To get a better understanding of the impacts of keywords and class weights on model performance, we determined test accuracy for classes depending on the number of training samples. As shown in Fig. 1, keywords consistently boost performance on the most rare classes (i.e. those will less than 50 training samples), while maintaining performance for well-represented classes. Class weights, on the other hand, boost performance for rare classes at the expense of performance for well-represented classes.

### B. Impact of Keywords on Model Scores

In classifying the site, subsite, and histology of cancer pathology reports, there is an expectation that the model will learn certain short phrases associated with each class. For example, the word “lung” should result in a high classification probability for the associated cancer site. To make this intuitive notion quantitative, we evaluated each trained model on all possible bigrams found in the training corpus. We then identified bigrams that resulted in the maximum model probability for each class.

**Fig. 2.**
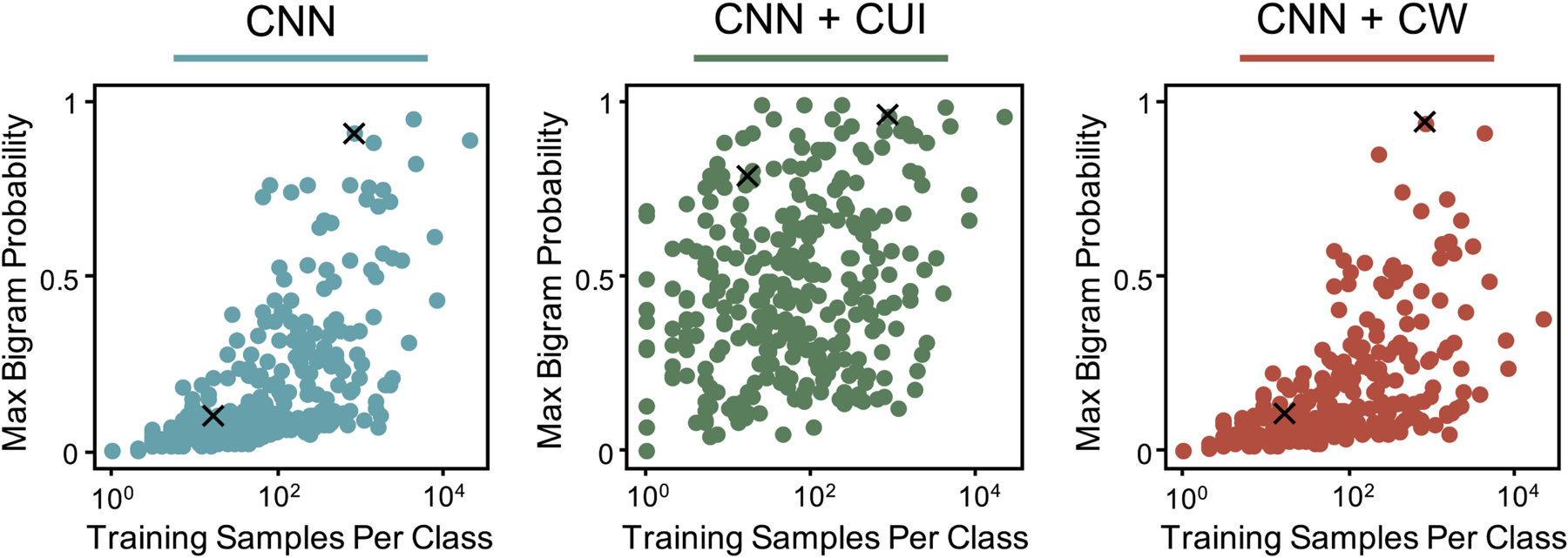
For each class in the subsite task, the maximum model output probability for all bigrams in the development training corpus is shown vs the number of training samples. The two X’s in each figure correspond to the example bigrams and scores shown in Tables IV–V.

As shown in Fig. 2, the addition of keywords (i.e. CUIs) has a large impact on the maximum scoring bigrams for each class of the subsite task. For the CNN model, classes with few training samples have a relatively low max bigram probability. For these classes, no single bigram in the entire training corpus generates a confident model classification. Intuitively, this is expected as the model must learn to fit large documents (i.e. thousands of tokens) with only a few labeled examples. The addition of keywords, however, enables the model to focus on specific bigrams without the need for many training samples. Notice that, although class weights boost macro performance, they have a negligible impact on the distribution of maximum bigram scores, showing that the keywords approach is qualitatively (and quantitatively) different.

To give a concrete example of associated bigrams for a given class, Table IV shows the top 5 bigrams for a rare subsite class C69.1 (Cornea, NOS). For the CNN and CNN + CW, the top bigrams are largely not specific to the subsite, but refer to the cancer site (C69 - Eye and Adnexa). Furthermore, the largest model output probability is approximately 0.1 for all possible bigrams. In contrast, the top bigrams for CNN + CUI all refer to the cornea and have a much higher model probability.

For a well-represented class, such as subsite C75.1 (Pituitary gland), the addition of keywords has much less impact on bigram probabilities. As shown in Table V, several of the top bigrams are in common across the models. The CNN is able to generate high bigram probabilities solely from the development training documents.

### C. Production Applications

Our results have shown that the addition of CUI based keywords improves model performance on rare classes and alters the model probabilities for short segments (i.e. bigrams). In many cases, however, CUIs or something similar may not be available to improve model performance. Therefore, to extend our strategy to enable production applications without previously generated keywords, we utilized normalized pointwise mutual information (NPMI) to determine keywords solely from the training corpus.

Here, we focus on a production scale dataset with over 4 million pathology reports. As shown in Table VI, even with a large corpus, the model still has difficulty with class imbalance, resulting in a low macro F1 score. Furthermore, CUI keywords continue to provide a substantial boost in macro F1 without much decrease to micro F1. Interestingly, the keywords provided by NPMI also improve macro with only a small drop in micro, substantially outperforming class weights for the histology task.

## IV Discussion

Recent work in deep learning for text classification tasks has largely focused on building better model architectures [2], [3], [16], [25]–[28]. Although model architecture is very important, our results suggest that a data centered (rather than model centered) approach may be useful as well. In the extreme case of a rare class with only one training sample, a model is confronted with a long sequence of tokens many of which may be unique to the given class. In this setting, model performance can be boosted through additional training keywords rather than attempts to modify the model architecture. Any future model can benefit from augmented training data including a collection of keywords associated with each class.

In a production setting, the keywords and short phrases can also serve as a mechanism to debug model errors. By determining the top bigrams based off of classification score for the model, a quick inspection can show if appropriate patterns are being mined from the data. In cases where model bigrams do not meet expectations (e.g. keywords are too generic), keywords can be introduced into training to increase user confidence in model classifications. To decrease the amount of manual involvement, there are already many approaches that can be used to generate possible keywords [20], [29]. Our results suggest that normalized pointwise mutual information, or a similar variant, can serve as a useful starting point to generate keywords. Keywords for under-performing classes could then be inspected and revised without the need to start from scratch. Furthermore, keyword inspection and annotation could be included within an active learning framework [30] to address class imbalance in a guided manner.

Supplying keywords during model training serves to provide a richer picture of the sample space, with long sequences of raw text showing realistic documents and short keyword phrases showing idealized class definitions. Given the amount of resources invested in debugging and tuning models in production settings, our results suggest a large return on investment for generating a set of keywords for each class. Furthermore, tracking and scoring bigrams for the model provides an efficient way for users to quantify model performance beyond typical measurements of loss and F1 score.

The use of keywords in the current approach can be viewed as a mechanism for guiding model training based on a known distribution of class scores for single tokens from the text. In this context, there is a natural comparison to Bayesian statistical modeling, with known keywords providing a prior distribution for conditional class probabilities. Generalizing the current results to include possible keyword distributions is an interesting topic for future investigation.

## V. Conclusion

Using a CNN model for text classification, we have shown that model performance on rare classes can be substantially improved by introducing keywords and shorts phrases for each class into the training set. For healthcare related applications, the keywords can be automatically extracted using UMLS CUIs, providing an automated solution to improve production applications with limited available training samples.

## Figures and Tables

**Fig. 3.**
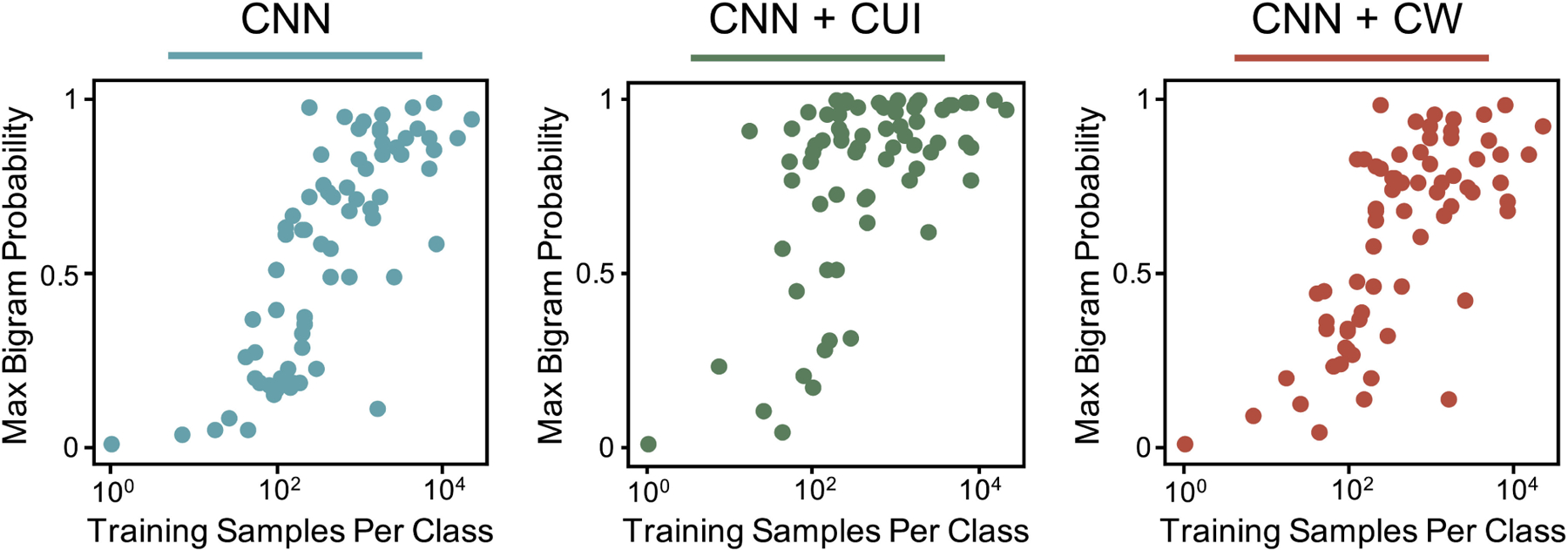
For each class in the site task, the maximum model output probability for all bigrams in the development training corpus is shown vs the number of training samples.

**Fig. 4.**
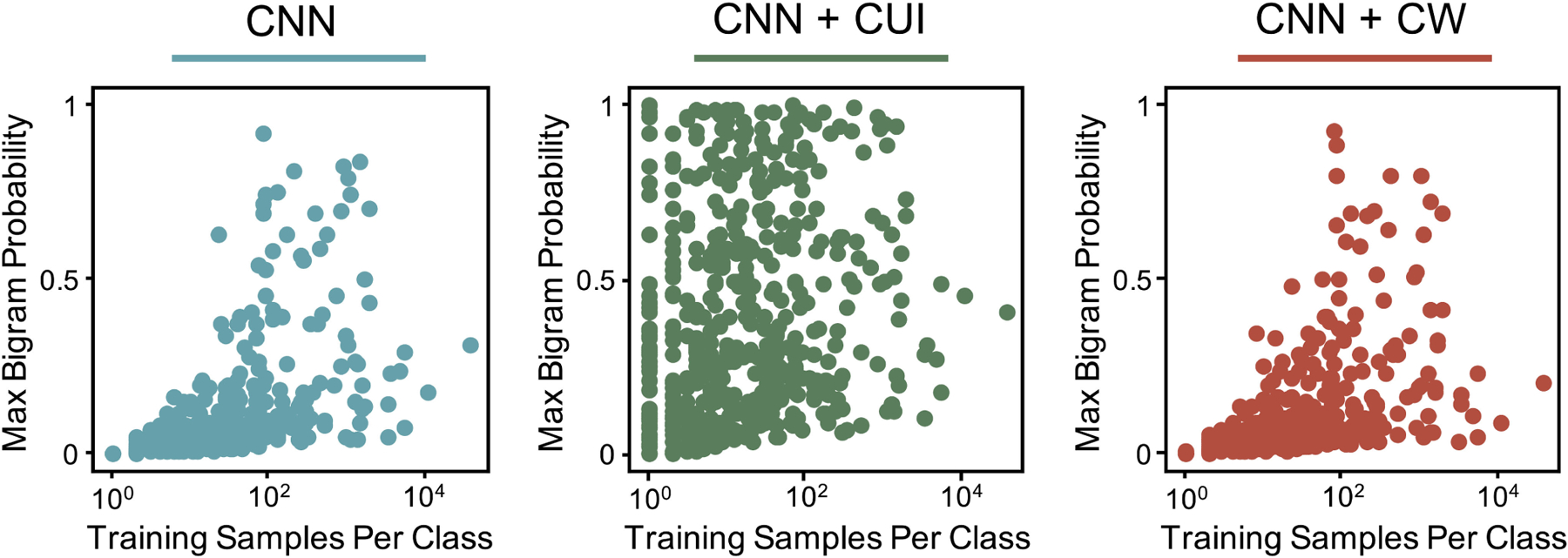
For each class in the histology task, the maximum model output probability for all bigrams in the development training corpus is shown vs the number of training samples.

**TABLE I:** Development Dataset Descriptions for Pathology Reports

Task	Train Docs	Val Docs	Test Docs	Unique Labels
Site	124289	26666	26230	70
Subsite	124289	26666	26230	315
Histology	124289	26666	26230	534

**TABLE II:** Production Dataset Descriptions for Pathology Reports

Task	Train Docs	Val Docs	Test Docs	Unique Labels
Site	3371508	579127	454307	70
Subsite	3371508	579127	454307	328
Histology	3371508	579127	454307	645

**TABLE III:** Test Micro and Macro F1 Scores for CNN on the Development Dataset of Pathology Reports

	CNN	CNN + CUIs	CNN + CW
Site Micro/Macro	93.62/69.81	93.67/71.31	93.51/72.07
Subsite Micro/Macro	78.22/36.74	78.46/39.65	77.40/38.35
Histology Micro/Macro	84.01/37.52	84.53/45.42	82.76/41.21

**TABLE IV:** Example Bigram Scores for a Rare Subsite Class (C69.1)

CNN		CNN + CUI		CNN + CW	
Bigram	Score	Bigram	Score	Bigram	Score
orbital eye	0.097	cornea eye	0.787	carcinoma eye	0.101
carcinoma eye	0.089	accompanied cornea	0.652	accompanied eye	0.089
melanoma eye	0.080	mass cornea	0.612	conjunctiva eye	0.084
cornea eye	0.077	neoplasm cornea	0.609	lid eye	0.079
orbit eye	0.075	lesion cornea	0.585	tumor eye	0.073

**TABLE V:** Example Bigram Scores for a Well-Represented Subsite Class (C75.1)

CNN		CNN + CUI		CNN + CW	
Bigram	Score	Bigram	Score	Bigram	Score
adenoma pituitary	0.912	adenoma pituitary	0.961	adenoma pituitary	0.943
gonadotroph pituitary	0.733	gonadotroph pituitary	0.945	origin pituitary	0.873
fluid pituitary	0.713	sellar pituitary	0.936	hormones pituitary	0.758
washing pituitary	0.705	pituitary gonadotroph	0.902	washing pituitary	0.739
mass pituitary	0.701	hormones pituitary	0.864	mass pituitary	0.722

**TABLE VI:** Test Micro and Macro F1 Scores for CNN Trained on Production Dataset With Over 4M Pathology Reports

	CNN	CNN + CUIs	CNN + CW	CNN + NPMI
Site Micro/Macro	92.82/70.79	92.82/71.46	92.46/71.51	92.79/71.04
Subsite Micro/Macro	69.99/34.80	70.14/38.34	69.37/37.77	69.78/37.71
Histology Micro/Macro	79.46/35.80	79.40/39.09	76.47/36.38	79.30/38.67

**TABLE VII:** Test Micro/Macro F1 Scores for CUI and NPMI Keywords With Fixed *α* = 1.0 and Varying *K* on the Development Dataset

	CNN + CUI			CNN + NPMI		
*K*	Site	Subsite	Histology	Site	Subsite	Histology
1	93.64/71.50	78.33/40.53	84.17/46.90	93.50/70.92	77.82/36.67	83.91/40.84
5	93.67/71.31	78.46/39.65	84.53/45.42	93.68/71.06	78.26/38.24	84.19/43.63
10	93.55/70.74	78.55/39.59	84.39/43.76	93.58/70.94	78.32/38.16	84.03/41.80
20	93.61/71.29	78.35/38.88	84.30/42.06	93.66/70.87	78.07/37.96	84.22/41.11

**TABLE VIII:** Test Micro/Macro F1 Scores for CUI and NPMI Keywords With Fixed *K* = 5 and Varying *α* on the Development Dataset

	CNN + CUI			CNN + NPMI		
*alpha*	Site	Subsite	Histology	Site	Subsite	Histology
0.25	93.60/70.43	78.31/38.10	84.35/42.00	93.71/71.43	78.14/38.41	84.01/42.12
0.5	93.73/71.45	78.55/39.21	84.54/43.46	93.71/71.35	78.02/38.20	84.10/42.82
1.0	93.67/71.31	78.46/39.65	84.53/45.42	93.68/71.06	78.26/38.24	84.19/43.63
2.0	93.63/71.31	78.41/39.47	84.45/45.66	93.61/70.94	77.95/37.47	83.89/41.30

**TABLE IX:** Test Micro/Macro F1 Scores for CW With Different Values of *μ* on the Development Dataset

	CNN + CW		
*μ*	Site	Subsite	Histology
0.05	93.48/71.88	77.17/37.74	83.22/40.71
0.15	93.51/72.07	77.40/38.35	82.76/41.21
1.0	93.59/71.46	77.97/38.15	83.08/39.63

## Footnotes

1 NJSCR is no longer in the SEER Program, but is included in the current data release.

## Appendix

### Maximum Bigram Probabilities for Site and Histology

In addition to the maximum model output probabilities shown for the subsite task, we generated similar figures for site (Fig. 3) and histology (Fig. 4). Similar to subsite, the histology task shows a drastic shift in the bigrams scores. Site also shows an increase in bigram scores, but does not have as many rare classes as subsite or histology.

### Hyperparameters

See Tables VII, VIII, IX.
